# Novel Radioligands for Cyclic Nucleotide Phosphodiesterase Imaging with Positron Emission Tomography: An Update on Developments Since 2012

**DOI:** 10.3390/molecules21050650

**Published:** 2016-05-19

**Authors:** Susann Schröder, Barbara Wenzel, Winnie Deuther-Conrad, Matthias Scheunemann, Peter Brust

**Affiliations:** Department of Neuroradiopharmaceuticals, Institute of Radiopharmaceutical Cancer Research, Helmholtz-Zentrum Dresden-Rossendorf, Permoserstraße 15, Leipzig 04318, Germany; s.schroeder@hzdr.de (S.S.); b.wenzel@hzdr.de (B.W.); w.deuther-conrad@hzdr.de (W.D.-C.); m.scheunemann@hzdr.de (M.S.)

**Keywords:** positron emission tomography, phosphodiesterases, cyclic nucleotide signaling, PDE inhibitors, PDE radioligands, imaging

## Abstract

Cyclic nucleotide phosphodiesterases (PDEs) are a class of intracellular enzymes that inactivate the secondary messenger molecules, cyclic adenosine monophosphate (cAMP) and cyclic guanosine monophosphate (cGMP). Thus, PDEs regulate the signaling cascades mediated by these cyclic nucleotides and affect fundamental intracellular processes. Pharmacological inhibition of PDE activity is a promising strategy for treatment of several diseases. However, the role of the different PDEs in related pathologies is not completely clarified yet. PDE-specific radioligands enable non-invasive visualization and quantification of these enzymes by positron emission tomography (PET) *in vivo* and provide an important translational tool for elucidation of the relationship between altered expression of PDEs and pathophysiological effects as well as (pre-)clinical evaluation of novel PDE inhibitors developed as therapeutics. Herein we present an overview of novel PDE radioligands for PET published since 2012.

## 1. Introduction

Positron emission tomography (PET) is a powerful imaging technique in nuclear medicine for non-invasive *in vivo* localization of radiolabeled molecular probes (radiotracers). PET enables the quantitative kinetic measurement of physiological and biochemical processes by means of radiotracers labeled with short-lived positron emitting radionuclides, like fluorine-18 (t_1/2_ = 109.8 min), carbon-11 (t_1/2_ = 20.4 min), nitrogen-13 (t_1/2_ = 9.98 min), and oxygen-15 (t_1/2_ = 2.03 min). Hence, this method allows visualization of metabolic and transport processes, protein biosynthesis, and binding of ligands to specific receptors or enzymes [1]. PET imaging is widely used for diagnosis and (pre-)clinical research in oncology, neurology and cardiology [2,3,4,5,6,7].

The enzyme class of cyclic nucleotide phosphodiesterases (PDEs) consists of 11 families which differ in their amino acid sequences and thus in their three-dimensional structures, regulatory properties, distribution within the organism, intracellular expression and subcellular localization, as well as substrate specificity. PDEs degrade the secondary messenger molecules cyclic adenosine monophosphate (cAMP) and cyclic guanosine monophosphate (cGMP) due to hydrolysis of the phosphodiester bond of the cyclic phosphate group [8,9,10]. The dual-substrate specific PDE families 1, 2, 3, 10, and 11 inactivate both cAMP and cGMP while PDEs 4, 7, and 8 are cAMP selective and PDEs 5, 6, and 9 specifically hydrolyze cGMP. Signal transduction mediated by the cyclic nucleotides affects various physiological processes like cell growth, differentiation and proliferation, metabolism, inflammation, and apoptosis [11,12,13,14,15]. The signaling cascades of cAMP and cGMP are affected by the PDEs altering the intracellular level of the respective cyclic nucleotide. Specific PDE inhibitors will raise the concentration of cAMP and/or cGMP and thus enhance cyclic nucleotide signaling. Hence, pharmacological inhibition of PDEs can provide a strategy for treatment of various diseases such as neurological, immune or inflammatory disorders, cancer, and heart diseases [9,14,16,17,18,19,20,21,22].

Therefore, appropriate PET radioligands for *in vivo* visualization and quantification of PDEs are of growing importance in clinical research to: (**1**) investigate the relationship between altered PDE expression and pathophysiological effects; and (**2**) provide a translational tool for evaluation of novel PDE inhibitors as therapeutics. The first PET radioligands described as imaging agents for a PDE enzyme were reported for the PDE4 family [23] and later found to be convenient for human application [24]. In the following ten years, development of further PET radioligands for the PDE families 1, 5, and 10 did not result in any suitable imaging agent for human use as extensively reviewed by Andrés *et al.* [25] in 2012. The herein presented overview is an update of the recently developed ^11^C- and ^18^F-labeled radioligands for PET imaging of the PDEs 2, 4, 5, 7, and 10. So far no radioligands have been reported for the PDE family subtypes 3, 6, 8, 9, and 11.

## 2. PDE2 Radioligands

Phosphodiesterase 2 (PDE2) is a member of the dual-substrate specific PDE family degrading both cAMP and cGMP [26]. The enzyme is encoded by the gene *PDE2A* and expressed in three isoforms. These isoforms differ in their N-terminal amino acid sequence and in their intracellular localization: PDE2A1 is a cytosolic protein, whereas PDE2A2 as well as PDE2A3 are membrane-associated [20,27,28]. Regarding enzyme kinetics, there are no differences known between the three isoforms [14]. PDE2A activity is regulated by allosteric binding of cGMP at the regulatory subunits that induces a conformational change of the catalytic domain resulting in accessible substrate binding sites in the active center [29,30,31,32].

The PDE2A protein is mainly expressed in the brain and only at low levels in certain peripheral tissues such as spleen, adrenal gland, heart, liver, kidney, lung, and small intestine [9,33,34,35,36]. In addition, this enzyme is expressed in particular tumors, for instance malignant melanoma cells [37,38], adrenocarcinoma [39], and mammary carcinoma [40]. In the brain, PDE2A is highly expressed in cortex, hippocampus, striatum, substantia nigra, globus pallidus, habenulae, bulbus olfactorius, tuberculum olfactorium, and amygdala [41,42]. The specific localization in structures of the limbic system indicates a modulation of important neuronal functions associated with emotion, learning, and memory [41,43,44,45,46,47]. Therefore, it is assumed that PDE2A is involved in the pathophysiology of neuropsychiatric and neurodegenerative disorders, such as depression and Alzheimer’s disease [42,48,49].

Pharmacological inhibition of PDE2A leads to an increase of the intracellular levels of cAMP and cGMP and is suggested to improve neuronal plasticity [43,49,50,51,52,53]. Thus, PDE2A inhibitors are considered as a promising approach for treatment of related neurological diseases [43,48,49,50,51,54]. Accordingly, development of specific PET radioligands for *in vivo* imaging and quantification of PDE2A has been recently gaining in importance in brain research (www.clinicaltrials.gov: NCT02584569).

**Figure 1:**
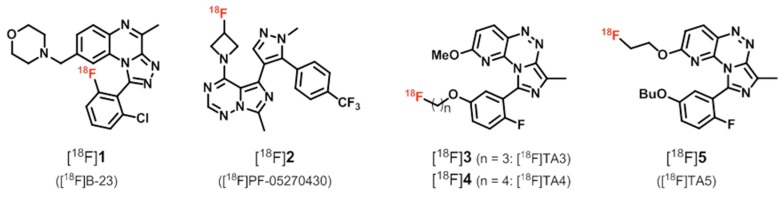
Molecular structures of ^18^F-labeled PDE2A inhibitors for PET.

**Scheme 1:**
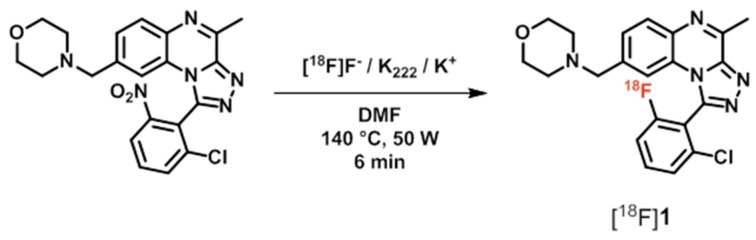
Radiosynthesis of [^18^F]**1**.

In 2013, the first two ^18^F-labeled PDE2A radioligands have been published by Janssen Pharmaceutica NV, [^18^F]**1** ([^18^F]B-23) [48,55], and Pfizer Inc., [^18^F]**2** ([^18^F]PF-05270430) [48,56] (Figure 1). The triazoloquinoxaline derivative **1** [48,55] is of high inhibitory potency towards the PDE2A protein, but of low PDE2A/PDE10A selectivity (IC_50_(PDE2A) = 1 nM; IC_50_(PDE10A) = 11 nM). Notably, the distribution pattern of PDE2A and PDE10A is comparable: both enzymes are highly expressed in the caudate nucleus as a part of the striatum [34]. For that reason, high selectivity of PET radioligands towards PDE2A is substantially for specific imaging of this enzyme. The ^18^F-labeled analogue [^18^F]**1** has been synthesized in a one-step nucleophilic aromatic radiolabeling strategy by using the related nitro precursor and K^+^/[^18^F]F^−^/K_2.2.2_-carbonate complex under microwave heating [55] (Scheme 1). In biodistribution and microPET imaging studies with rats, the highest accumulation of [^18^F]**1** at 2 min post injection has been observed in striatum followed by hippocampus, cortex, and cerebellum [55]. However, the reported specific uptake in the striatum may be caused by binding of [^18^F]**1** to PDE2A and PDE10A in this brain region as a result of its low selectivity. Additionally, polar radiometabolites have been detected in the brain with 4% of total activity at 2 min up to 18% at 10 min post injection [55]. In conclusion, [^18^F]**1** appears not to be an appropriate radioligand for *in vivo* imaging of the PDE2A protein.

**Scheme 2:**
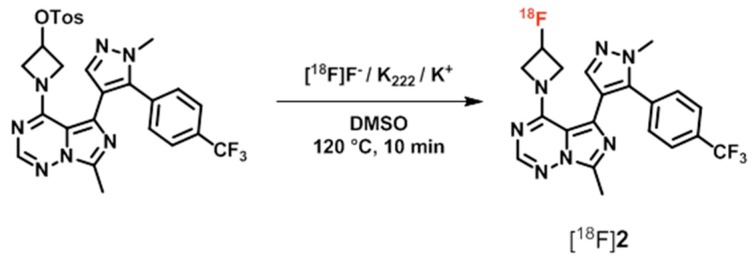
Radiosynthesis of [^18^F]**2**.

The PDE2A inhibitor **2** (PF-05270430) [48,56] has been developed out of a series of imidazolotriazine compounds and is of high potency and selectivity towards PDE2A (IC_50_(PDE2A) = 0.5 nM; IC_50_(PDE10A) > 3000 nM) with good brain uptake. The radioligand [^18^F]**2** has been prepared initially by nucleophilic aliphatic displacement of the tosylate group of the corresponding precursor with tetra-*n*-butylammonium [^18^F]fluoride in *tert*-amyl alcohol [56]. In order to increase the radiochemical yield of [^18^F]**2** from < 2% [56] up to 20%, an optimized radiosynthesis procedure by using K^+^/[^18^F]F^−^/K_2.2.2_-carbonate complex in dimethyl sulfoxide under GMP-compliant conditions has been developed by Morley *et al.* [57] (Scheme 2). In PET studies on monkeys a rapid and high uptake of [^18^F]**2** in striatum and low uptake in cerebellum has been reported, consistent with the distribution pattern of the PDE2A in brain [35,41]. Furthermore, no defluorination has been observed and an effective blocking of radioligand accumulation in the striatum has been described [56]. Notably, [^18^F]**2** has already been evaluated in first human PET studies in healthy volunteers as reported in a conference abstract in 2013 [58] and very recently in a detailed publication by Naganawa *et al.* [59]. The results of these studies indicate that [^18^F]**2** is suitable for PET imaging of PDE2A in the human brain based on the observed radioligand accumulation in PDE2A-specific brain regions, the favorable kinetic profile, the high tolerability and safety as well as the good metabolic stability [59].

Besides [^18^F]**1** and [^18^F]**2**, three ^18^F-labeled imidazopyridotriazine derivatives as potential PDE2A radioligands for PET, [^18^F]**3**, [^18^F]**4**, and [^18^F]**5** ([^18^F]TA3–5, Figure 1), have been developed in our group [60,61,62] based on a patented lead compound [43].

**Scheme 3:**
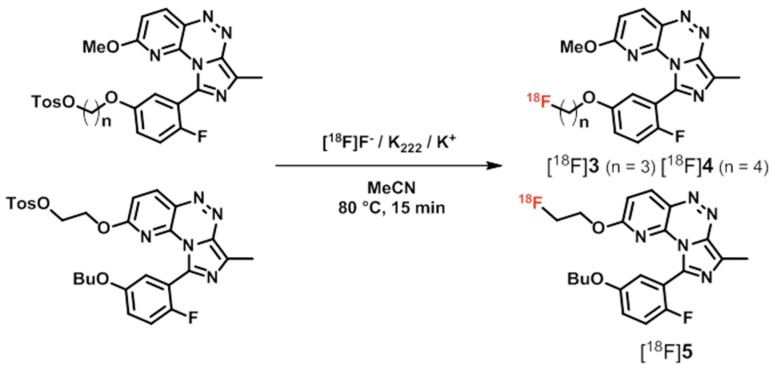
Radiosyntheses of [^18^F]**3**, [^18^F]**4**, and [^18^F]**5**.

The non-radioactive fluoroalkoxyphenyl derivatives **3** and **4** are of high inhibitory potency and selectivity towards PDE2A (**3**: IC_50_(PDE2A) = 11 nM; IC_50_(PDE10A) = 318 nM; **4**: IC_50_(PDE2A) = 7 nM; IC_50_(PDE10A) = 913 nM) [61]. In addition, replacement of the methoxy group at the pyridinyl moiety of the lead compound by a fluoroethoxy side chain resulted in the most potent derivative out of this series, compound **5**, with a significantly increased PDE2A/PDE10A selectivity (IC_50_(PDE2A) = 3 nM; IC_50_(PDE10A) > 1000 nM) [62]. The radioligands [^18^F]**3**, [^18^F]**4**, and [^18^F]**5** have been prepared in a one-step radiosynthesis by nucleophilic aliphatic substitution of the tosylate group of appropriate precursors with the anhydrous K^+^/[^18^F]F^−^/K_2.2.2_-carbonate complex [61,62] (Scheme 3). *In vitro* autoradiography on rat brain slices displayed region-specific binding of [^18^F]**3** and [^18^F]**4** [61,62], which is consistent with the PDE2A distribution pattern in rat brain [34,41] and could be blocked by co-incubation with the lead compound. In autoradiographic studies with [^18^F]**5** on sections of rat and pig brain, a homogenous and non-displaceable distribution of activity has been detected indicating high non-specific binding of this radioligand (unpublished work). Small-animal PET studies with [^18^F]**3** in mice showed a fast wash out of activity from the striatum while a constantly increased uptake in the non-target region cerebellum has been observed [61,62]. This finding likely reflects the accumulation of brain penetrating radiometabolites which has been confirmed in metabolism studies with [^18^F]**3**, [^18^F]**4**, and [^18^F]**5** where 29%, 4% [61], and 10%, respectively, of total activity in the mouse brain have been represented by the intact radioligands at 30 min post injection. It is suggested that cytochrome P450 enzyme induced metabolic degradation of the ^18^F-bearing alkyl side chains in [^18^F]**3**–**5** resulted in formation of the corresponding brain penetrating ^18^F-alkyl alcohols, aldehydes or carboxylic acids [61,63,64].

Overall, we have concluded that none of the up to now generated ^18^F-labeled imidazopyrido- triazine derivatives meets the requirements for PET imaging of PDE2A in the brain. Nevertheless, further structural modification of these promising radioligands is currently in progress and might result in metabolically stable derivatives.

## 3. PDE4 Radioligands

Up to now, phosphodiesterase 4 (PDE4) is one of the most studied PDE families. This enzyme specifically hydrolyses cAMP and is expressed by four genes *PDE4A*, *B*, *C*, and *D* that generate overall more than 20 isoforms [65,66]. The different genes vary in their coding sequences for the C- and N-terminus of the enzyme and the numerous isoforms are characterized by distinct regulatory subunits [9,20,35,67]. In general, PDE4 is expressed in a wide range of tissues and cell types, such as brain, smooth muscle, heart, lung, kidney, endothelium and immune cells [9,20,34,67]. In the periphery, PDE4B and PDE4D are the most abundant PDE4 mRNAs with highest levels of PDE4B in spleen, lung, and bladder and of PDE4D also in bladder as well as skeletal muscle, and thyroid [34]. Regarding mRNA levels in brain, PDE4B is the most abundantly expressed PDE4, with highest levels in hypothalamus, followed by PDE4A and PDE4D. By comparison, PDE4C shows the lowest expression in both peripheral and brain tissues [34,68].

PDE4 activity is suggested to be involved in several processes such as brain function primarily associated with mood changes [69], monocyte [70] and macrophage activation [71], neutrophil infiltration [72], and vascular smooth muscle proliferation [73]. Thus, PDE4 inhibitors are of great interest for treatment of neurological disorders, including depression, Alzheimer’s disease, and disorders of the immune and inflammatory system, particularly chronic obstructive pulmonary disease and asthma [9,20,32,67,68,74]. The main drawback of the early developed PDE4 inhibitors has been emesis as one side effect of these compounds because of low selectivity towards the different PDE4 isoforms [9,32,67]. To overcome this problem, second generation compounds, like cilomilast, have been identified with reduced side effects liability [75].

**Figure 2:**
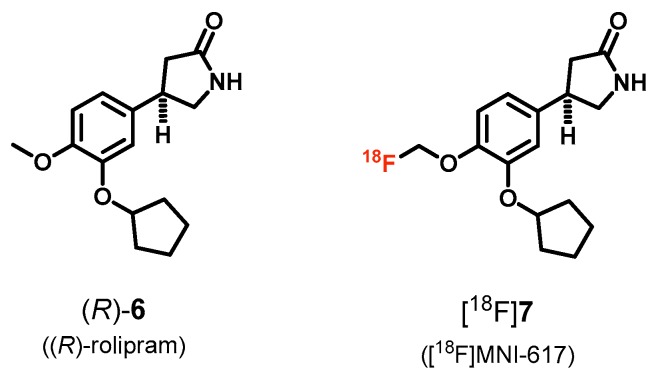
Molecular structures of PDE4 inhibitor (*R*)-**6** and the ^18^F-analogue thereof for PET.

The most extensively studied PDE4 inhibitor **6** (rolipram, Figure 2) leads to an increase in cerebral cAMP levels and is known as potent antidepressant but side effects have hampered clinical application [20,67]. It has been reported that rolipram inhibits the PDE4A, B, C and D isoforms at a similar degree [76], while other studies showed differences [77,78]. However, the ^11^C-labeled analogue of (*R*)-**6** ([^11^C]rolipram) [79] has been suggested as a promising PET radioligand for *in vivo* evaluation of PDE4 levels and alterations in cAMP signaling pathways [24] as already reviewed elsewhere [25,67]. Meanwhile, [^11^C]**6** has successfully been used to demonstrate downregulation of PDE4 in major depressive disorder [80] and to monitor progression of certain cardiac disorders such as heart failure, mitral valve disease, diabetic cardiomyopathy, and adriamycin cardiotoxicity [81].

Very recently, Thomae *et al.* [82,83] reported on the development of structurally modified derivatives of (*R*)-**6** with the aim to generate ^18^F- and ^123^I-labeled PDE4 radioligands for PET or SPECT application, respectively. In general, low tolerance of PDE4 for structural changes in (*R*)-**6** has been stated as well as a significant decrease in PDE4 affinity for the iodo derivatives that consequently have been suggested to be not suitable as SPECT radioligands. Out of this series, the fluorinated analogue **7** (MNI-617, Figure 2) has been described as the most promising compound with a five-fold increased PDE4 affinity over (*R*)-**6** (*K*_D_ = 0.26 nM *vs.* 1.6 nM) and thus has been selected for ^18^F-labeling [82,83].

**Scheme 4:**
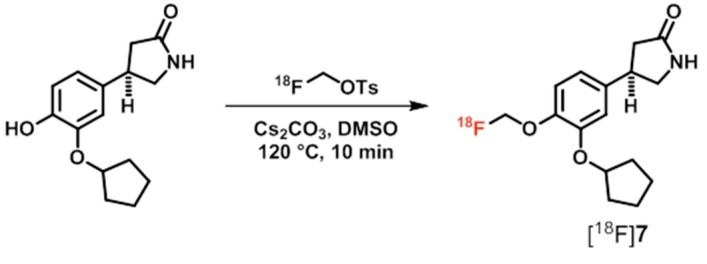
Radiosynthesis of [^18^F]**7**.

Initially, [^18^F]**7** has been prepared in an one-pot two-step radiosynthesis procedure via ^18^F-labeling of diiodomethane using K^+^/[^18^F]F^−^/K_2.2.2_-carbonate complex followed by *O*-alkylation of the corresponding phenol precursor with the resulting [^18^F]fluoroiodomethane [82]. At the 21st International Symposium on Radiopharmaceutical Sciences, Columbia 2015, the group has presented an optimized two-step radiolabeling strategy by first generating [^18^F]fluoromethane tosylate that has been used as a prosthetic group for subsequent *O*-alkylation of the phenol precursor under basic conditions (Scheme 4). In this radiosynthesis, the overall radiochemical yield of [^18^F]**7** has been increased from 1.5% up to 29%. In PET studies on monkeys a fast and high uptake of [^18^F]**7** in the brain has been observed, with specific distribution in regions of known PDE4 expression [82,83]. Although no data concerning the metabolic stability of [^18^F]**7** have been published yet, the authors stated that the [^18^F]fluoromethyl group might be defluorinated *in vivo* leading to bone uptake of the resulting free [^18^F]fluoride [83] which is indeed a known process in the metabolism of ^18^F-alkylated radioligands. Thus, the group also synthesized and evaluated the deuterated analogue D_2_-[^18^F]**7** (D_2_-[^18^F]MNI-617) [83,84]. PET studies in monkeys with D_2_-[^18^F]**7** revealed a comparable brain distribution and kinetic profile like [^18^F]**7**. However, accumulation of activity in the skull indicates fast defluorination of D_2_-[^18^F]**7** [83]. Despite the high and PDE4-specific brain uptake as well as the favorable brain kinetics of [^18^F]**7** and D_2_-[^18^F]**7**, it has been concluded that these radioligands are not suitable for PET imaging of PDE4 because of their low metabolic stability [83].

## 4. PDE5 Radioligands

The cGMP specific phosphodiesterase 5 (PDE5) is a cytosolic protein activated by allosteric binding of cGMP [85,86]. This enzyme is encoded by the gene *PDE5A* and is expressed as three isoforms, PDE5A1, PDE5A2, and PDE5A3 that differ in their N-termini. It is assumed that PDE5A expression is regulated by the specific promotor sequences of these isoforms [87]. PDE5A1 and PDE5A2 are widely distributed in numerous tissues such as bladder, lung, stomach, thyroid, pancreas, heart, intestine, vascular smooth muscle and at low levels in brain, whereas PDE5A3 is specifically expressed in vascular smooth muscle [9,34,87]. It is well known that PDE5A regulates vascular smooth muscle contraction by controlling the intracellular cGMP level, especially in penis and lung. Thus, pharmacological inhibition of PDE5A activity is in clinical application for treatment of erectile dysfunction and pulmonary arterial hypertension (PAH) using **8** (sildenafil, Viagra™), **9** (vardenafil, Levitra™), tadalafil (Cialis™), and avanafil (Stendra™) [9,20,32,88,89,90]. Additionally, an increased myocardial expression of PDE5A in advanced heart failure has been reported [91]. Hence, PDE5A inhibitors are also suggested to prevent or reverse cardiac hypertrophy and adverse cardiac remodeling and to have cardioprotective effects against ischemia or reperfusion injury [9,91,92,93,94].

**Figure 3:**
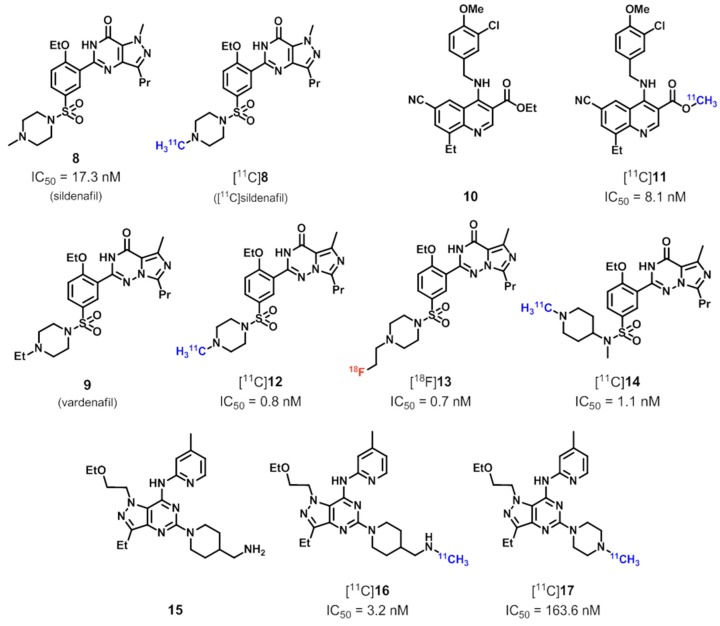
Molecular structures of PDE5A inhibitors and ^11^C- or ^18^F-labeled derivatives thereof for PET.

**Scheme 5:**
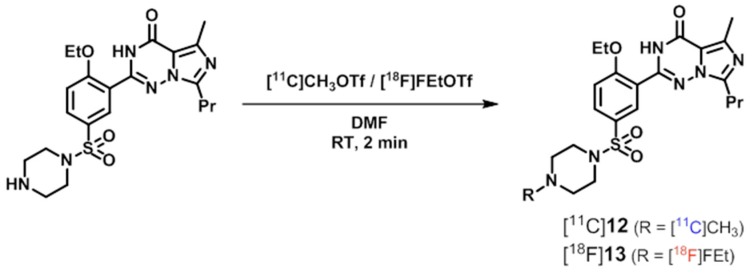
Radiosyntheses of [^11^C]**12** and [^18^F]**13**.

**Scheme 6:**
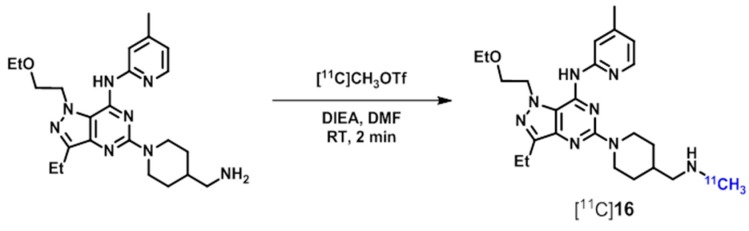
Radiosynthesis of [^11^C]**16**.

Chekol *et al.* [25,95,96,97] developed a series of ^11^C- and ^18^F-labeled derivatives of specific PDE5A inhibitors known from literature [97,98,99] for *in vivo* quantification of PDE5A in pulmonary vasculature and visualization of PDE5A expression in myocardium via PET (Figure 3). Most of the PDE5A radioligands have been prepared by *N*-alkylation of the corresponding amine precursors using [^11^C]methyl trifluoromethanesulfonate or the 2-[^18^F]fluoroethyl analogue (Scheme 5 and Scheme 6 for [^11^C]**12**, [^18^F]**13**, and [^11^C]**16**). In the case of [^11^C]**11**, radiolabeling has been performed by esterification starting from a carboxylic acid precursor [97]. The retention of these radiolabeled derivatives in lung, which is the tissue with most abundant PDE5A expression [34,87,89], has been the main criterion for their suitability as radioligands for PET imaging of this enzyme [97].

In biodistribution studies in mice, low accumulation in lung has been observed for the ^11^C-labeled sildenafil [^11^C]**8**, the 4-benzylaminoquinoline derivative [^11^C]**11**, and the ethoxyethyl pyrazolopyrimidine-based [^11^C]**17** followed by high to moderate uptake of [^11^C]**16** and the radio- labeled vardenafil derivative [^11^C]**14** [97]. Out of this series, the most potent derivatives [^11^C]**12** and [^18^F]**13** displayed the highest accumulation in lung with standard uptake values (SUVs) of 8.0 and 8.9, respectively, at 30 min post injection [97]. Furthermore, [^11^C]**12** showed a 40-fold increased uptake in heart of transgenic mice overexpressing cardiac specific PDE5 [25,95]. Blocking studies have been performed for [^11^C]**12** and [^11^C]**16** using the specific PDE5A inhibitor tadalafil at which accumulation of the radioligands in lung could be reduced significantly (SUV = 8.0 *vs.* 1.0 and 6.6 *vs.* 3.6) [97]. In conclusion, the radiolabeled vardenafil derivatives [^11^C]**12** and [^18^F]**13** as well as compound [^11^C]**16** are recommended as appropriate radioligands for *in vivo* imaging of PDE5A protein and thus have been selected for further biological investigation by the authors [97].

Besides the pharmacological interest of inhibiting PDE5A activity in peripheral disorders, expression of this enzyme in specific brain regions like cerebellum, hippocampus, substantia nigra, medulla, and thalamus indicate an important role of PDE5A in the central nervous system associated with neuroinflammatory and neurodegenerative processes as well as cognition [34,100,101,102]. It has been demonstrated that PDE5 inhibitors have neuroprotective effects in different animal models of neurological diseases [103,104,105,106]. In particular, benefits of **8** (sildenafil) in cognition deficits have been observed in transgenic mouse models of Alzheimer’s disease [107,108]. However, it was unclear whether **8** readily crosses the blood-brain barrier [109]. With regard to that, PET studies with [^11^C]**8** in rats have been performed [109], but this radioligand displayed low brain uptake (SUV = 0.18 at 30 min p.i.), homogenous distribution and negligible specific binding as a result of metabolic degradation probably caused by *N*-dealkylation and formation of [^11^C]CH_3_OH. However, quantification of **8** and cGMP levels in the cerebrospinal fluid of non-human primates clearly proved that this PDE5 inhibitor is brain penetrating after oral administration at a therapeutic dose suggesting its indication for treatment of Alzheimer’s disease [109].

**Scheme 7:**
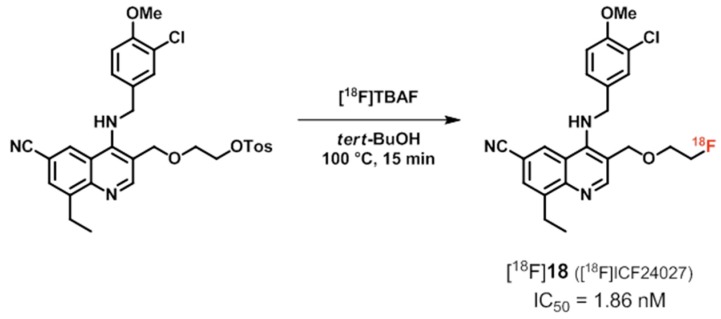
Molecular structure and radiosynthesis of [^18^F]**18** as PDE5A radioligand for PET (TBAF = tetra-*n*-butylammonium fluoride).

Up to now, no successful detection of PDE5 in the brain for quantification of its expression or occupancy has been reported. Very recently, the ^18^F-labeled quinoline-based radioligand [^18^F]**18** ([^18^F]IC24027, Scheme 7) for PET imaging of PDE5A in the brain has been developed by researchers from the Institut national de la santé et de la recherche médicale (INSERM), Clermont-Ferrand, France, in cooperation with our group [110]. Radiosynthesis of [^18^F]**18** has been performed by nucleophilic aliphatic substitution of the tosylate group of the corresponding precursor using tetra-*n*-butylammonium [^18^F]fluoride in *tert*-butanol (Scheme 7).

*In vitro* autoradiographic studies on porcine brain slices revealed highest binding densities of [^18^F]**18** in the substantia nigra followed by corpus callosum which is consistent with the known PDE5A distribution pattern in human brain tissue [111]. However, [^18^F]**18** binding was only slightly reduced by co-incubation with the non-radioactive reference compound and **8** (sildenafil) indicating a moderate specific binding of this radioligand. That may be a result of the low PDE5 expression in the brain with only nanomolar density [112] and therefore, a radioligand with subnanomolar PDE5 potency is needed for quantification of this enzyme in brain. *In vivo* metabolism studies in mice showed fast degradation of [^18^F]**18** with only 7% of intact radioligand in plasma and 12%–15% in brain at 30 min post injection, respectively. Two brain penetrating radiometabolites have been observed [110]. In conclusion, [^18^F]**18** is not suitable for PET neuroimaging of PDE5 because of the insufficient specificity of binding and the low metabolic stability. Nevertheless, this outcome will be helpful for our ongoing work in the development of appropriate ^18^F-labeled quinoline derivatives for visualization and quantification of the PDE5 enzyme in brain with PET.

## 5. PDE7 Radioligands

Phosphodiesterase 7 (PDE7) is a cAMP-specific enzyme encoded by the two genes *PDE7A* and *PDE7B*, and is expressed in three cytosolic isoforms [9,20,34] that differ in their coding sequences for the C- and N-terminus. In contrast to other PDE isoforms, the N-termini of PDE7A and PDE7B are of unknown function and do not contain any of the regulatory subunits so far identified in PDEs [9,20,35]. PDE7A is expressed at low levels in the brain and at high levels in spleen, heart, skeletal muscle and several immune cells, for example T lymphocytes [113]. By contrast, PDE7B is dominant in the central nervous system with most abundant expression in striatal brain regions such as caudate nucleus, caudate putamen, and nucleus accumbens followed by cortical tissues and hippocampus while in the periphery the highest PDE7B levels are found in bladder and heart [9,33,34,35,114,115].

On the basis of these expression patterns, specific PDE7 inhibitors are suggested as promising approach for treatment of inflammatory and neurological disorders associated with T-cell activation and function [116], Parkinson’s disease [117], and addiction [118,119,120]. However, very little is known about the physiological effects of PDE7 activity to date. With regard to elucidation of the role of this enzyme in several neurological diseases, appropriate PET radioligands would enable *in vivo* imaging and quantification of the PDE7 protein in brain.

**Figure 4:**
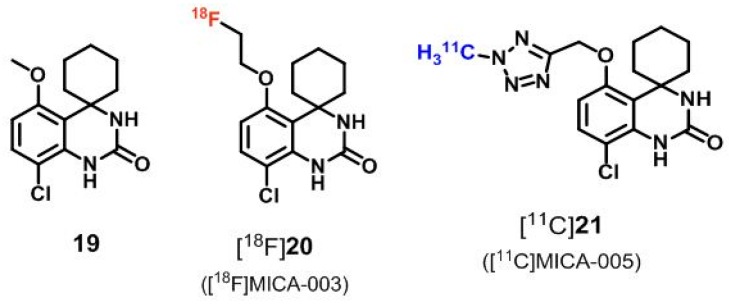
Molecular structures of PDE7A/B inhibitor **19** and ^18^F- or ^11^C-labeled derivatives thereof for PET.

In 2015, Thomae *et al.* [121,122] reported on the development of the first two radiolabeled derivatives for that purpose, [^18^F]**20** ([^18^F]MICA-003) and [^11^C]**21** ([^11^C]MICA-005), based on a series of spiroquinazolinones as potent dual PDE7A/B inhibitors, like compound **19** [123] (Figure 4).

**Scheme 8:**
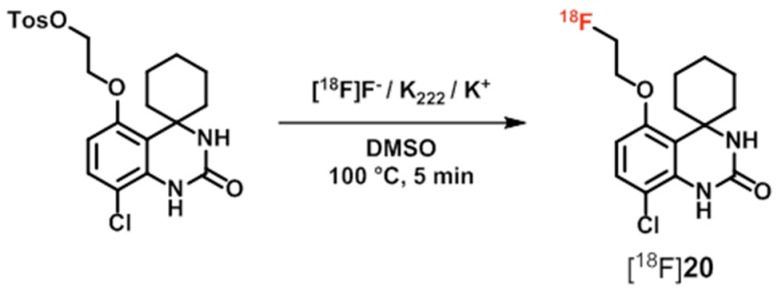
Radiosynthesis of [^18^F]**20**.

**Scheme 9:**
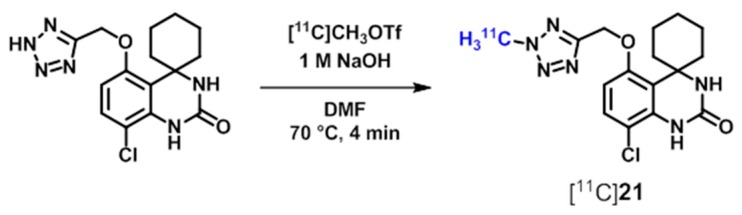
Radiosynthesis of [^11^C]**21**.

The spirocyclic compounds **20** and **21** are of high inhibitory potencies towards PDE7 with IC_50_ values of 17.0 nM and 1.7 nM, respectively [121,122]. The ^18^F-labeled analogue [^18^F]**20** has been prepared by nucleophilic aliphatic substitution of the tosylate group of the corresponding precursor using K^+^/[^18^F]F^−^/K_2.2.2_-carbonate complex (Scheme 8). A two-step procedure has been performed for the radiosynthesis of [^11^C]**21** by methylation of the tetrazole precursor with [^11^C]methyl triflate under basic conditions (Scheme 9) [121,122]. Biodistribution studies with [^18^F]**20** in mice revealed a high brain uptake (4%–5%ID/g) at 5 min post injection besides the highest accumulation of this radioligand in heart (10%–11%ID/g) [122]. In microPET imaging studies with [^18^F]**20** and [^11^C]**21**, both radioligands displayed rapid uptake and homogenous distribution in the mouse brain followed by a fast wash out [121,122]. For [^18^F]**20** the SUV in brain reached a plateau after 27 min [122] and that, together with the observed non-specific distribution of the radioligands, has been suggested as a result of radiometabolite accumulation in the brain. This presumption has been confirmed by *in vivo* metabolite analysis demonstrating only 74% and 85% of intact [^18^F]**20** and [^11^C]**21** in mouse brain at 5 min post injection, respectively [121,122]. For [^18^F]**20**, the brain penetrating radiometabolite has been identified as 2-[^18^F]fluoroethanol by HPLC analysis using the non-radioactive 2-fluoroethanol as reference [122]. Finally, the radioligands [^18^F]**20** and [^11^C]**21** are suggested to be unfavorable for *in vivo* quantification of PDE7 in the brain via PET, and development of more stable spiroquinazolinone derivatives is currently in progress [122].

## 6. PDE10 Radioligands

The dual-substrate specific phosphodiesterase 10 (PDE10) degrades both cAMP and cGMP [124]. The enzyme is encoded by the gene *PDE10A* and expressed in 18 splice variants with cytosolic PDE10A1 and membrane-associated PDE10A2 as the major isoforms in humans [125,126,127]. Allosteric binding of cAMP activates the enzyme resulting in an increased cAMP degradation at competitive inhibition of cGMP hydrolysis [128,129]. The expression of PDE10A is distinctive and most abundant in striatal brain areas, in particular in medium spiny neurons, with highest levels in caudate nucleus and nucleus accumbens, and at much lower levels in other brain regions [124,130,131]. In peripheral tissues, PDE10A is expressed in testes and to a lower extent for example in the thyroid gland, kidneys, heart, and lung [124,131]. Due to the high PDE10A density in striatum, this enzyme is suggested to play an important role in the pathophysiology of neurodegenerative and neuropsychiatric disorders such as Huntington’s disease, Parkinson’s disease, and schizophrenia [132,133,134,135,136]. Thus, PDE10A inhibitors are of great interest for treatment of the mentioned brain disorders as reflected by the large number of compounds developed during the last decade [134,136,137,138]. With regard to that, it is not surprising that PDE10A specific radioligands are also of growing importance for quantification and visualization of this enzyme *in vivo* using PET.

### 6.1. Radioligands Structurally Related to the PDE10A Inhibitor MP-10

**Figure 5:**
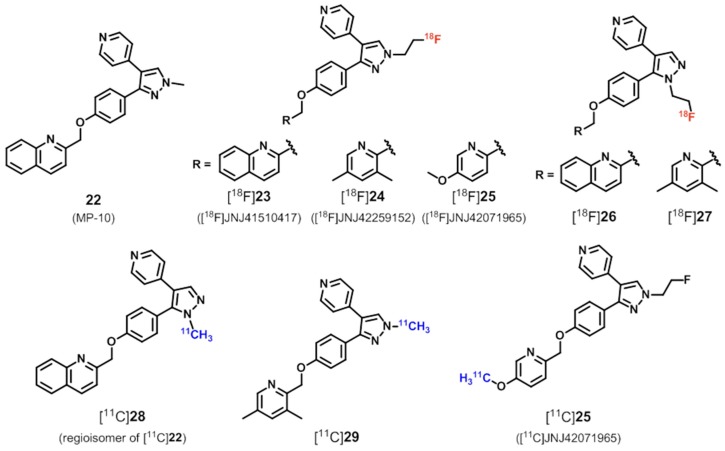
Molecular structures of PDE10A inhibitor **22** and ^11^C- or ^18^F-labeled derivatives thereof for PET.

Based on the highly potent PDE10A inhibitor **22** (MP-10, IC_50_ = 1.26 nM [139]; IC_50_ = 0.37 nM [140]; IC_50_ = 0.18 nM with 100-fold selectivity over other PDEs [141]; IC_50_ = 0.02 nM [142]; IC_50_ = 0.64 nM [143]) and the radiolabeled analogue, [^11^C]**22** ([^11^C]MP-10) [141,144,145,146,147], a series of structurally related ^11^C- and ^18^F-labeled derivatives have been developed for PET imaging of PDE10A as partly reviewed in 2012 by Andrés *et al.* [25,143,144,148,149,150,151] (Figure 5).

Briefly, in baseline microPET imaging and biodistribution studies in rats and PDE10A knockout mice [^18^F]**23** ([^18^F]JNJ41510417, IC_50_ = 0.5 nM [149]) showed PDE10A-specific and reversible binding, but rather slow brain kinetics [25,143,148,149]. The PDE10A radioligands [^18^F]**24** ([^18^F]JNJ42259152, IC_50_ = 2 nM [151]; IC_50_ = 1.58 nM [143]) and [^18^F]**25** ([^18^F]JNJ42071965, IC_50_ = 3.16 nM [143]) are characterized by more favorable kinetic profiles and high striatum-to-cerebellum ratios of 5.4 and 4.4 at 30 min post injection, respectively [143,150,151]. Furthermore, PET studies in healthy humans with [^18^F]**24** showed appropriate radioligand distribution, brain kinetics, and safety [152,153], and thus, this radioligand has been further evaluated in patients with Huntington’s disease (HD) [154]. In this exploratory study, a significant loss of striatal PDE10A levels compared to healthy controls has been observed [154]. Additionally, PET studies with [^18^F]**24** in a mouse model of HD provided evidence for early regional dysfunctions in PDE10A signaling, involving the caudate putamen and lateral globus pallidus [155]. Moreover, the suitability of [^18^F]**24** PET for assessment of the pharmacological interaction of dopamine neurotransmission and PDE10A availability in rats has been reported very recently [156]. 

Notably, [^18^F]**23**, [^18^F]**24**, and [^18^F]**25** all undergo (**1**) oxidative metabolic cleavage of the phenolic ether bond, as also described for [^11^C]**22** [141,147], and (**2**) *N*-dealkylation at the pyrazolo site. This degradation results in the formation of two brain penetrating radiometabolites: (**1**) the related phenol [141] and (**2**) 2-[^18^F]fluoroethanol or its oxidation products [25,143,149,150,151,153]. Despite this, it has been stated that PDE10A can be reliably quantified in the human brain by PET using [^18^F]**24** [153].

In 2014, Ooms *et al.* [143] published a comparative biological study of several derivatives which are structurally based on the above mentioned radioligands (Figure 5). Radiosyntheses of [^18^F]**26** and [^18^F]**27**, [^11^C]**28**, [^11^C]**29**, and [^11^C]**25** have been performed by using [^18^F]fluoroethyl bromide and [^11^C]methyl iodide or triflate in dimethylformamide at 90 °C under basic conditions [25,143,150]. Baseline microPET imaging in rats with [^18^F]**26**, [^18^F]**27**, and [^11^C]**29** revealed no specific accumulation in striatum due to low PDE10A potency [143,150]. In comparison to that, the radioligand [^11^C]**28** (IC_50_ = 3.47 nM [143]) showed slightly higher striatal uptake, but blocking studies in rats and PET imaging in PDE10A knockout mice displayed off target binding in the brain [143]. Finally, [^11^C]**25** exhibited the most favorable brain kinetics as well as the highest specific accumulation in striatum within this series of radioligands structurally related to compound **22** [143]. However, detection of brain penetrating radiometabolites with up to 12% of total activity in cerebellum at 30 min post injection [143] might limit the application of [^11^C]**25**, especially for *in vivo* quantification of PDE10A in the brain via PET.

**Figure 6:**
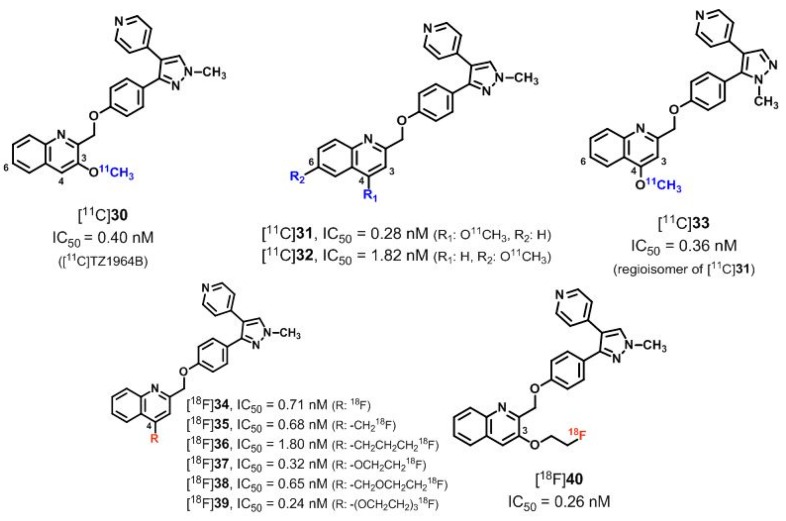
Molecular structures of further ^11^C- or ^18^F-labeled derivatives of compound **22** as PDE10A radioligands for PET.

Recently, Fan *et al.* [157] and Li *et al.* [158] reported on the development of further ^11^C- and ^18^F-labeled derivatives (Figure 6) out of a small library of pyrazolo-*N*-methyl regioisomers of compound **22** (MP-10) with different substitution patterns at the 2-methylquinoline moiety [158,159].

**Scheme 10:**
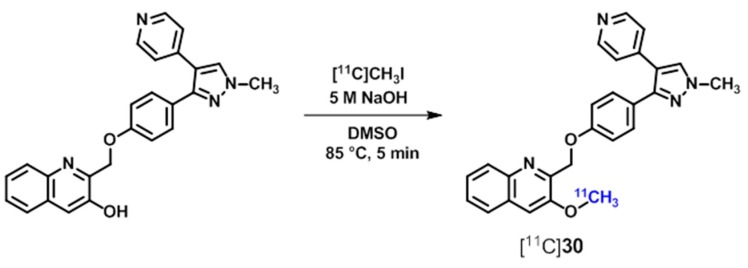
Radiosynthesis of [^11^C]**30**.

The 3-, 4-, and 6-methoxy derivatives **30** (TZ1964B), **31**, and **32** as well as the 4-methoxy regioisomer **33** are of high potency (Figure 6) and selectivity towards PDE10A [157,159]. The radioligands [^11^C]**30**–**33** have been prepared by methylation of the corresponding quinolinol precursors [158] with [^11^C]methyl iodide under basic conditions [157] (Scheme 10 for [^11^C]**30**). Out of this series, [^11^C]**30** and the most potent derivative [^11^C]**31** showed the highest brain uptake in rats with striatum-to-cerebellum ratios of 6.0 and 4.5 at 60 min post injection, respectively [157]. MicroPET studies in non-human primates with [^11^C]**30** and [^11^C]**31** revealed a high and specific accumulation of both radioligands in striatum. Metabolic degradation of [^11^C]**31** occurred significantly faster compared to [^11^C]**30** with 42% and 65% of the intact radioligands in plasma at 60 min post injection [157]. Further evaluation of [^11^C]**30** ([^11^C]TZ1964B) and its tritiated analogue [^3^H]**30** ([^3^H]TZ1964B) in rats and non-human primates [160] revealed high PDE10A-specific and reversible binding. In conclusion, [^11^C]**30** has been stated as the most promising candidate for *in vivo* quantification and visualization of PDE10A in the brain via PET because of its high brain uptake and striatum-to-cerebellum ratio, good striatal retention, favorable metabolic stability as well as advantageous brain kinetics [157].

**Scheme 11:**
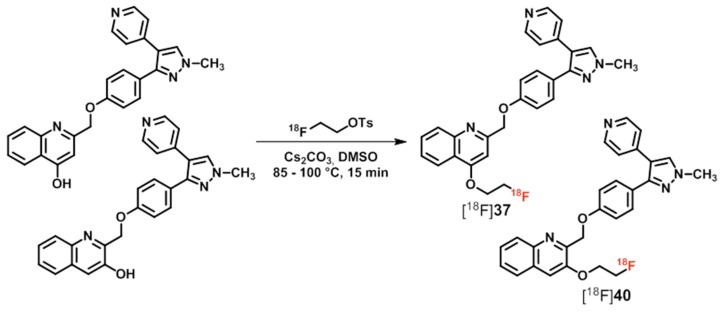
Radiosyntheses of [^18^F]**37** and [^18^F]**40**.

Out of a series of related fluorine-containing quinoline derivatives, **34**–**40** (Figure 6) displayed high potency and selectivity towards PDE10A with IC_50_-values in a range of 0.24 to 1.80 nM [158]. Most of the corresponding radioligands have been prepared in one-step labeling procedures by (**1**) direct nucleophilic halogen exchange for [^18^F]**34** and [^18^F]**35**, and (**2**) aliphatic substitution via the related tosylate or mesylate precursors for [^18^F]**36**, [^18^F]**38**, and [^18^F]**39**. A two-step labeling strategy has been performed for the radiosyntheses of [^18^F]**37** and [^18^F]**40** using 2-[^18^F]fluoroethyl tosylate [158] (Scheme 11).

In biodistribution studies in rats, all of these radioligands showed comparable or even higher brain uptake than [^11^C]**22** ([^11^C]MP-10). However, [^18^F]**34**–**36** have failed due to an increasing accumulation of activity in bone during the 60-min scan indicating metabolic defluorination of these radioligands. The 4- or 3-fluoroethoxy derivatives [^18^F]**37** and [^18^F]**40** have been identified as the most promising radioligands based on ≥ 2-fold striatum-to-non-target (cerebellum or cortex) ratios at 30 min post injection, fast clearance from non-target regions and specific binding in the PDE10A- enriched striatum [158]. Thus, [^18^F]**37** and [^18^F]**40** have been further evaluated in non-human primates. MicroPET imaging studies displayed high accumulation of both radioligands in caudate and putamen with striatum-to-non-target ratios > 3 at 40 min post injection as well as enhanced washout kinetics over [^11^C]**22** [158]. Metabolism studies at 30 min post injection revealed > 90% and 73% of intact [^18^F]**37** or [^18^F]**40** in plasma while two polar radiometabolites for each radioligand have been detected, and further validation of [^18^F]**37** and [^18^F]**40** is in progress to clarify the suitability of these derivatives for PET imaging of PDE10A in the brain [158].

**Figure 7:**
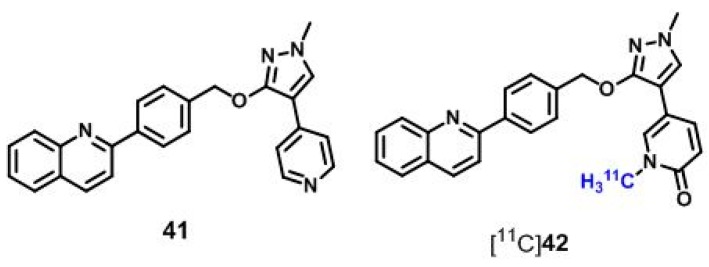
Molecular structures of PDE10A inhibitor **41** and the ^11^C-labeled derivative thereof for PET.

In 2015, Hamaguchi *et al.* [161] reported on the development of another series of compound **22**-related derivatives based on the quinoline analogue **41** [162] (Figure 7). Compared to **22** (MP-10), compound **41** is of reduced inhibitory potency towards PDE10A (IC_50_ = 29 nM *vs.* 0.55 nM [161,162]), but of higher metabolic stability as shown by *in vitro* studies using mouse and human liver microsomes (M/HLM) [162]. Structural modification of **41** led to the most promising derivative **42** that exhibited a significantly increased PDE10A potency (IC_50_ = 5.1 nM) and further enhanced *in vitro* stability in M/HLM studies [161]. Thus, **42** has been selected for ^11^C-labeling to evaluate this novel PDE10A inhibitor *in vivo* with PET (Figure 7).

**Scheme 12:**
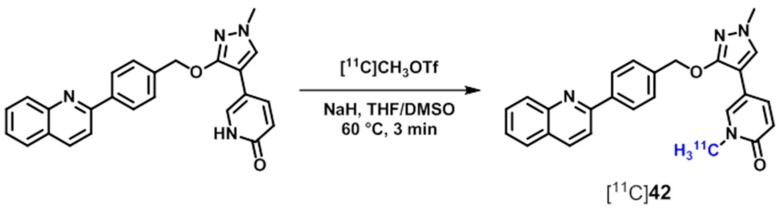
Radiosynthesis of [^11^C]**42**.

The radioligand [^11^C]**42** has been prepared by methylation of the corresponding pyridinone precursor using [^11^C]methyl triflate under basic conditions [161] (Scheme 12). Biodistribution studies in mice revealed a significantly higher accumulation of [^11^C]**42** in striatum than in cerebellum with SUVs of 0.6 and 0.2 at 60 min post injection, respectively [161]. PET imaging studies in rats displayed specific uptake of [^11^C]**42** in striatum that could be blocked after pre-treatment with an excess of **22** (10 mg/kg). Hence, [^11^C]**42** has been stated to show good brain penetration and high specificity of binding in rodents [161]. These preliminary results indicate the suitability of [^11^C]**42** for PET imaging of PDE10A in the brain. However, further data concerning *in vivo* metabolism studies and dosimetry have not been published yet.

### 6.2. Radioligands Structurally not Derived from the PDE10A Inhibitor MP-10

**Figure 8:**
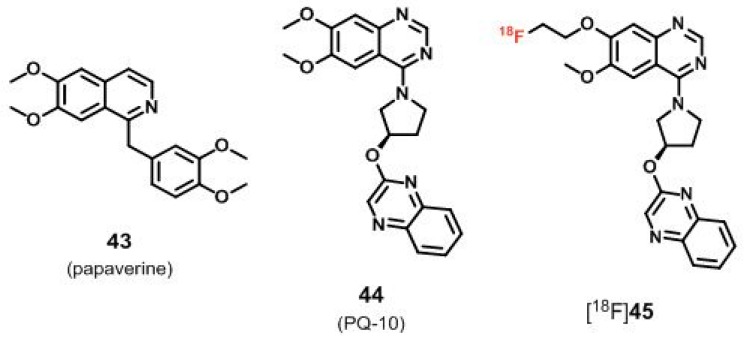
Molecular structures of **43**, a related PDE10A inhibitor and the ^18^F-labeled derivative thereof for PET.

To possibly overcome the problem of metabolic stability of derivatives related to the scaffold of compound **22** (MP-10), our group generated the structurally different radioligand [^18^F]**45** [163,164] (Figure 8) for PET imaging of PDE10A that has already been mentioned by Andrés *et al.* [25]. Based on the potent papaverine-related PDE10A inhibitor **44** (PQ-10, K_i_ = 4 nM [165]; IC_50_ = 16 nM [166]) published by Pfizer [165] (Figure 8), the novel corresponding ^18^F-labeled fluoroethyl derivative [^18^F]**45** has been developed [164,166].

*In vitro* autoradiography on rat brain slices displayed region-specific binding of [^18^F]**45** which corresponds with the distribution pattern of PDE10A in rat brain [130]. However, non-displaceable binding of [^18^F]**45** in particular brain regions of known PDE3A expression [167] has been observed. Therefore, it is assumed that [^18^F]**45** additionally binds to PDE3A as indicated by the non-negligible inhibitory potency of this radioligand towards PDE3A (IC_50_ = 88.7 nM [168]) [164]. Biodistribution studies in mice displayed insufficient brain uptake as well as low specific binding of [^18^F]**45**. Metabolism studies revealed 70% of intact [^18^F]**45** in mouse plasma at 60 min post injection whereas a single lipophilic radiometabolite has been detected [164]. In the brain, a very high fraction of intact [^18^F]**45** has been observed with 94% and 93% at 30 and 60 min post injection, respectively. In conclusion, [^18^F]**45** has been stated to be unsuitable for quantitative PET imaging of PDE10A due to its low specificity [164].

**Figure 9:**
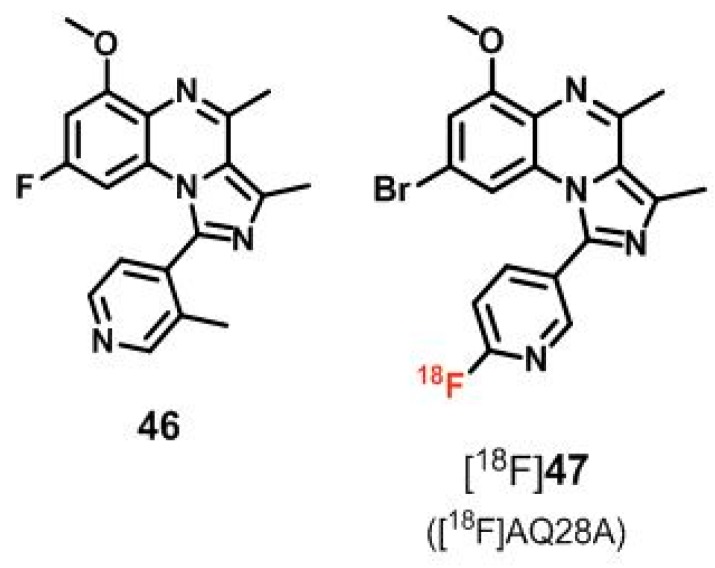
Molecular structures of a PDE10A inhibitor and the ^18^F-labeled derivative thereof for PET.

Consequently, further development of appropriate PDE10A radioligands in our group was based on an alternative structural approach using 1-arylimidazo[1,5-*a*]quinoxaline **46** (Figure 9) as lead compound [169]. Out of a small library of 1- and 8-(2′-fluoro)pyridine-substituted derivatives of **46** [170,171], compound **47** (AQ28A, Figure 9) showed high potency as well as best selectivity towards PDE10A (IC_50_ = 2.95 nM and > 1000 nM for all other PDEs [171]) and has been selected for ^18^F-labeling [170,172,173].

**Scheme 13:**
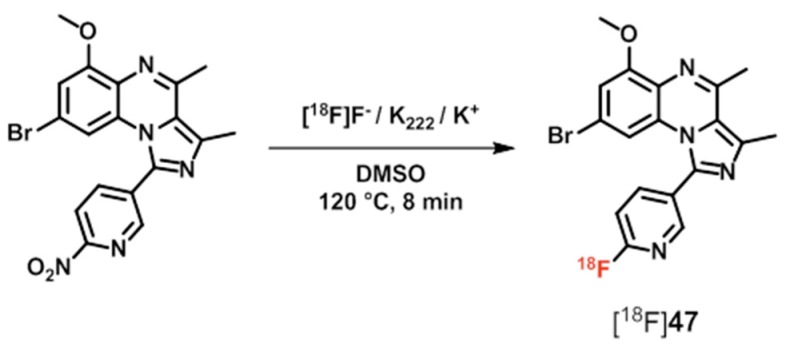
Radiosynthesis of [^18^F]**47**.

[^18^F]**47** has been prepared initially by direct nucleophilic halogen exchange using the related bromo precursor and the K^+^/[^18^F]F^−^/K_2.2.2_-carbonate complex in dimethyl sulfoxide [170,173]. To increase the radiochemical yield of [^18^F]**47**, an optimized nucleophilic aromatic radiosynthesis procedure starting from the corresponding nitro precursor has been developed [172,173] (Scheme 13). *In vitro* autoradiography on mouse, rat and pig brain slices displayed region-specific binding of [^18^F]**47** [172,173], which corresponds to the known PDE10A distribution pattern in brain of various mammalian species [130,131] and could also be detected by *ex vivo* autoradiographic studies in mouse brain at 30 min post injection. In microPET imaging studies in mice, a high initial brain uptake has been observed (SUV = 1.8 at 3 min p.i.) with peak accumulation of [^18^F]**47** in striatum (SUV = 2.7 at 4 min p.i.) followed by a fast wash out. Additionally, a striatum-to-cerebellum ratio of 1.4 at 30 min post injection and a 60% reduced striatal uptake after pre-treatment with **22** indicate specific binding of [^18^F]**47** *in vivo*. Metabolism studies in mice revealed 35%–57% of intact [^18^F]**47** in plasma and 71% in brain, respectively, at 30 min post injection [173]. Formation of two brain penetrating radiometabolites has been observed which are slightly more polar than [^18^F]**47** and thus might result from metabolic hydroxylation or *O*-demethylation [169]. However, *in vivo* blocking studies and *ex vivo* autoradiography revealed no specific binding of these radiometabolites either to target- or to non-target regions [173]. Therefore, [^18^F]**47** might be appropriate for quantification of PDE10A in the brain.

Notably, although there is evidence that PDE10A is involved in the regulation of whole body energy balance [174], to the best of our knowledge, PDE10A expression associated with obesity has not been analyzed so far. Hence, we used [^18^F]**47** in PET/MR studies in various mouse models of obesity with the aim to quantify PDE10A not only in striatum but also in brown adipose tissue (BAT) [172]. In brief, follow-up studies after high fat diet revealed significantly increased uptake of [^18^F]**47** in BAT and striatum with 130% and 30% higher SUVs compared to controls, respectively [172]. Based on these preliminary results we hypothize that PDE10A might be a novel therapeutic target for treatment of obesity [172]. Nevertheless, further biological evaluation is needed and currently in progress to verify the suitability of [^18^F]**47** as specific PDE10A imaging agent for PET.

From 2012 to 2015, further ^11^C- and ^18^F-labeled radioligands for PET imaging of PDE10A that are structurally different from compound **22** (MP-10) have been reported by several groups and will be discussed in the following section.

**Figure 10:**
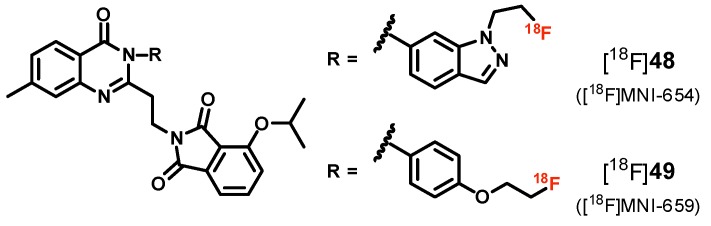
Molecular structures of ^18^F-labeled PDE10A radioligands for PET.

The radioligands published by Barret *et al.* [175,176], [^18^F]**48** and [^18^F]**49** ([^18^F]MNI-654 and [^18^F]MNI-659, Figure 10), are of high PDE10A binding affinity with *K*_D_-values of 0.029 nM and 0.097 nM [176], respectively.

**Scheme 14:**
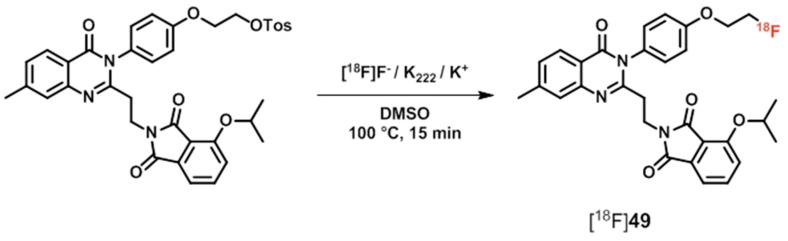
Radiosynthesis of [^18^F]**49**.

[^18^F]**48** and [^18^F]**49** have been prepared by nucleophilic aliphatic substitution of the tosylate group of the corresponding precursors using the K^+^/[^18^F]F^−^/K_2.2.2_-carbonate complex [176] (Scheme 14 for [^18^F]**49**). In preliminary PET studies in non-human and human primates, a region-specific brain uptake of both radioligands paralleling the expected PDE10A distribution and a dose-dependent occupancy of PDE10A by blockade with **22** have been observed [175]. Thus, the authors stated that [^18^F]**48** and [^18^F]**49** might be suitable for PET imaging of PDE10A in the brain. Subsequent *in vivo* evaluation in healthy humans revealed moderate metabolic stability of both radioligands and a similar degradation profile with about 20% of intact [^18^F]**48** and [^18^F]**49** in plasma at 120 min post injection [176]. In brain distribution studies, a high and specific accumulation of both radioligands in the PDE10A-rich regions caudate, putamen, and globus pallidus has been observed. In these brain areas, [^18^F]**48** and [^18^F]**49** showed SUVs of 0.7–1.0 and 1.5–2.5, respectively, at 60 min post injection [176], and [^18^F]**49** displayed a much faster overall washout than [^18^F]**48**. Based on the more favorable brain kinetics and dosimetry of [^18^F]**49**, this radioligand has been selected for further assessment in patients with Huntington’s disease (HD) [177]. Results of this pilot study revealed that PET imaging with [^18^F]**49** is of high sensitivity and reliability for basal ganglia PDE10A and can clearly detect pathological loss of striatal PDE10A making it a promising biomarker for longitudinal studies in HD [177]. This assumption has been confirmed in a corresponding PET study in patients with pre-manifest and manifest HD (stage 1 and 2) that revealed significantly reduced [^18^F]**49** uptake in putamen and caudate nucleus compared to healthy controls [178]. In follow-up studies, about one year later, a more reduced striatal uptake of [^18^F]**49** has been observed in the HD patients indicating further decreased PDE10A levels, especially in the caudate nucleus [178], but evaluation in a larger HD cohort is needed to confirm these findings [178]. Nevertheless, the previous studies clearly demonstrate the suitability of [^18^F]**49** for imaging and quantification of PDE10A in the brain via PET as well as for assessment of the enzyme occupancy of novel PDE10A inhibitors developed as therapeutics (www.clinicaltrials.gov: NCT02001389).

**Figure 11:**
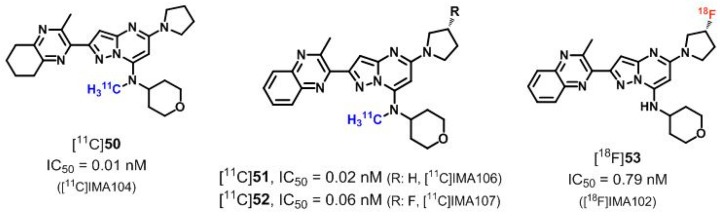
Molecular structures of ^11^C- or ^18^F-labeled PDE10A radioligands for PET.

On the basis of a pyrazolopyrimidine scaffold, Plisson *et al.* [142] reported on the development of three ^11^C- and one ^18^F-labeled derivatives that have been selected based on the inhibitory potencies of the related non-radioactive compounds **50**–**53** towards PDE10A (Figure 11).

**Scheme 15:**
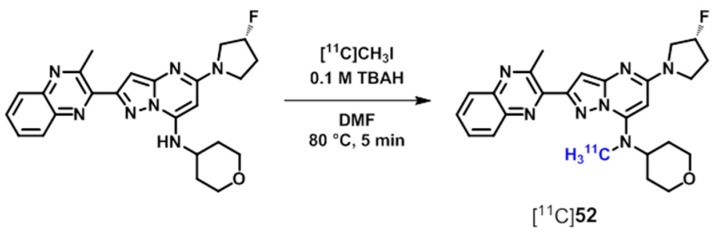
Radiosynthesis of [^11^C]**52** (TBAH = tetra-*n*-butylammonium hydroxide).

The radioligands [^11^C]**50** ([^11^C]IMA104), [^11^C]**51** ([^11^C]IMA106), and [^11^C]**52** ([^11^C]IMA107) have been prepared by *N*-alkylation of the corresponding secondary amine precursors with [^11^C]methyl iodide under basic conditions [142] (Scheme 15 for [^11^C]**52**). Radiosynthesis of [^18^F]**53** ([^18^F]IMA102) has been achieved by nucleophilic aliphatic substitution of the mesylate group of the related precursor using the K^+^/[^18^F]F^−^/K_2.2.2_-carbonate complex in dimethyl sulfoxide at 120 °C [142]. PET studies in pigs displayed heterogeneous brain distribution of all these radioligands with highest accumulation in striatum. However, the derivative [^11^C]**50** showed slow brain kinetics and [^18^F]**53** exhibited a low striatum-to-rest of brain ratio. Thus, [^11^C]**51** and [^11^C]**52** have been selected for further PET studies in baboons, but in contrast to the previous results in pigs, no washout of [^11^C]**51** from PDE10A-rich brain regions has been observed during the 120-min scan. Also [^11^C]**52** showed similar brain distribution but slightly slower kinetics in baboons than in pigs with peak uptake at 35 min post injection [142]. Both radioligands exhibited comparable moderate metabolic stability with 56% and 50% of intact [^11^C]**51** and [^11^C]**52** in baboon plasma at 90 min post injection [142]. The detected radiometabolites have been stated to be more polar than [^11^C]**51** or [^11^C]**52** and suggested not to be brain penetrating [142].

In blocking studies in pigs and baboons, specific binding of [^11^C]**52** in PDE10A-rich brain regions has been confirmed using **53** and compound **22** (MP-10) [142]. Hence, [^11^C]**52** ([^11^C]IMA107) has been progressed to human PET studies in healthy volunteers [142] resulting in a consistent regional brain distribution and reversible kinetics that indicates a good translation of the radioligand behavior between all species studied. These initial results provided evidence that [^11^C]**52** is appropriate for PET imaging of PDE10A because of good brain penetration, peak uptake in putamen at ~ 25 min (SUV ~ 2.5) and no adverse or clinically detectable pharmacologic effects [142]. In PET studies with [^11^C]**52** in patients with Huntington’s disease, an altered PDE10A expression was detectable early before symptomatic onset [179]. This radioligand has also been used to demonstrate striatal and pallidal loss of PDE10A expression, which is associated with duration and severity of motor symptoms and complications in Parkinson’s disease [180]. Very recently, further studies with [^11^C]**52** in patients with chronic schizophrenia have been reported [181]. Interestingly, these PET imaging studies revealed no significant differences in [^11^C]**52** uptake in the PDE10A-rich brain regions caudate, putamen, globus pallidus, thalamus, nucleus accumbens, and substantia nigra compared to healthy controls. Hence, the authors concluded that there is no evidence for an altered PDE10A level in patients with schizophrenia [181]. Furthermore, a clinical trial with the PDE10A inhibitor **22** (MP-10) in patients with an acute exacerbation of schizophrenia (www.clinicaltrials.gov: NCT01175135) revealed no differences between subjects that received compound **22** and the placebo control group [181].

These findings clearly show the general importance of PET imaging of PDE10A, and thus of appropriate radioligands for that purpose, to further investigate and understand the complex processes in probably related neuropsychiatric pathologies and the potential of PDE10A inhibitors as therapeutics.

**Scheme 16:**
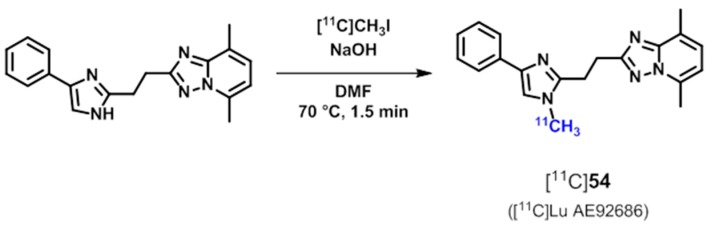
Molecular structure and radiosynthesis of [^11^C]**54** as PDE10A radioligand for PET.

The radioligand [^11^C]**54** ([^11^C]Lu AE92686, Scheme 16), published by Kehler *et al.* [182,183], has been selected as a promising candidate for PET imaging of PDE10A based on the favorable *in vitro* and *in vivo* properties of the non-radioactive compound [184], such as high inhibitory potency towards PDE10A (IC_50_ = 0.46 nM [183]; IC_50_ = 0.32 nM [184]; IC_50_ = 0.39 nM with > 1000-fold selectivity over other PDEs) and brain uptake (brain-to-plasma ratio in mice = 0.47) [182]. [^11^C]**54** has been prepared by *N*-alkylation of the corresponding imidazole precursor using [^11^C]methyl iodide under basic conditions [182,183] (Scheme 16).

Metabolism studies revealed fast *in vivo* degradation in rats and non-human primates with only 15% and 27% of intact [^11^C]**54** in plasma at 40 and 30 min post injection, respectively [182]. In rat brain, detection of a small amount of polar radiometabolites has been reported. PET studies with [^11^C]**54** in non-human primates displayed rapid brain uptake with highest accumulation in striatum and a fast overall wash out indicating a favorable kinetic profile. Specificity of [^11^C]**54** binding has been confirmed by pre-treatment with compound **22** (MP-10) resulting in a considerably dose-dependent decrease in striatal uptake [182]. Further evaluation of [^11^C]**54** in human PET studies revealed a comparable brain distribution with peak uptake in striatum at 40 min post injection (SUV ~ 4) that slowly decreased afterwards. Notably, a significantly slower metabolism with about 70% of intact radioligand in plasma at 60 min post injection was observed [182]. In conclusion, [^11^C]**54** seems to be suitable for PET imaging and quantification of PDE10A in the human brain. 

**Figure 12:**
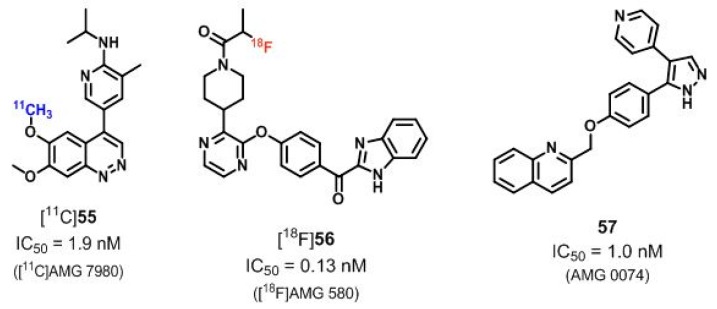
Molecular structures of ^11^C- or ^18^F-labeled PDE10A radioligands for PET and the PDE10A inhibitor **57**.

Hwang *et al.* [185,186,187] reported on the development of [^11^C]**55** and [^18^F]**56** ([^11^C]AMG 7980 and [^18^F]AMG 580, Figure 12) which have been selected as potential PET radioligands because of their high inhibitory PDE10A potencies, and in particular [^18^F]**56** because of its extraordinary selectivity (IC_50_ > 30 µM for other PDEs). Furthermore, high to very high binding affinities of the corresponding tritiated analogues towards PDE10A have been estimated with a *K*_D_-value of 0.94 nM for [^3^H]**55** in rat striatal homogenate [188] and *K*_D_-values of 51.7 pM, 71.9 pM, and 83.1 pM (male) or 84.1 pM (female) for [^3^H]**56** in rat, baboon, and human striatal tissues, respectively [187].

**Scheme 17:**
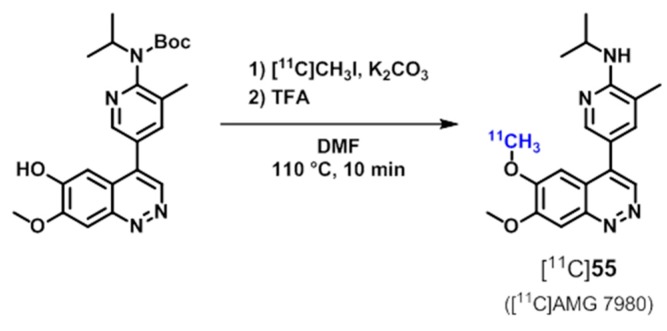
Radiosynthesis of [^11^C]**55**.

**Scheme 18:**
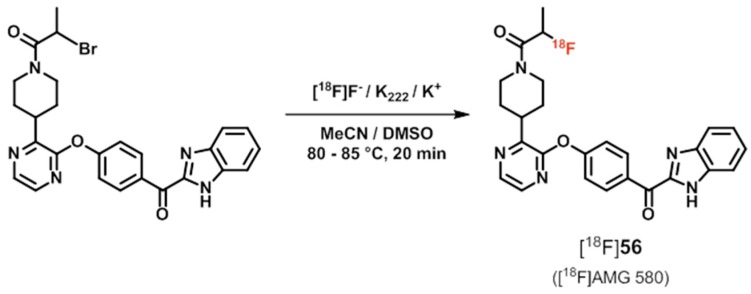
Radiosynthesis of [^18^F]**56**.

The radioligand [^11^C]**55** has been prepared in a one-pot two-step radiosynthesis by ^11^C-labeling of the *N*-*tert*-butoxycarbonyl (BOC)-substituted phenol precursor using [^11^C]methyl iodide followed by acidic hydrolysis to remove the protecting group [185] (Scheme 17). Radiosynthesis of [^18^F]**56** has been performed by direct nucleophilic aliphatic halogen exchange using the corresponding bromo precursor and the K^+^/[^18^F]F^−^/K_2.2.2_-carbonate complex [187] (Scheme 18).

Characterization of [^11^C]**55** in non-human primates revealed fast metabolism with 33% of intact radioligand in plasma at 30 min post injection. Related PET imaging studies displayed distribution of [^11^C]**55** throughout the brain with highest accumulation in PDE10A-specific regions like putamen and globus pallidus followed by a fast wash out [185]. The striatal uptake of [^11^C]**55** decreased in a dose-dependent manner after pre-treatment with a non-specified PDE10A inhibitor indicating moderate specific binding [185]. The authors suggested this might be a result of the fast dissociation rate of [^11^C]**55** as determined in the *in vitro* binding assay with [^3^H]**55** (dissociation half-life = 0.1 min [188]) [186].

By contrast, the related (*R*)- and (*S*)-enantiomers of **56** exhibited dissociation half-lives of 3.1 and 0.8 min [187,188]. *In vitro* autoradiography on rat, baboon, and human brain slices displayed a high and specific striatal accumulation of [^3^H]**56** that is consistent with the known PDE10A expression pattern [34,130,131] and could be blocked significantly by co-incubation with an excess of the PDE10A inhibitor **57** (AMG 0074, Figure 12) [187]. In microPET studies with [^18^F]**56** in rats, rapid brain penetration and region-specific uptake have been observed with highest accumulation in striatum (SUV = 2.77 at ~ 50 min p.i.), as well as moderate to low uptake in nucleus accumbens (SUV = 0.94) and cerebellum (SUV = 0.58) [187]. Based on these results, [^18^F]**56** has been further evaluated in non-human primates [186]. Metabolism studies in baboons and rhesus monkeys revealed fast *in vivo* degradation of [^18^F]**56** with approximately 25% or 35% of intact radioligand in plasma, respectively, at 30 min post injection. Corresponding PET studies displayed high accumulation of [^18^F]**56** in striatum and low uptake in other brain regions comparable to the previous findings in rats [186]. Specificity of [^18^F]**56** binding *in vivo* has been confirmed by pre-treatment with compound **22** (MP-10), but significantly reduced accumulation in the target regions has been obtained only at a larger excess of **22** (3 mg/kg) [186]. Furthermore, the PET images revealed higher levels of whole brain uptake in rhesus monkeys that might indicate higher non-specific binding and/or a slower wash out of [^18^F]**56** compared to that in baboons.

In conclusion, these *in vivo* studies do not clearly verify whether [^11^C]**55** or [^18^F]**56** might be suitable for PET imaging of PDE10A in the brain. Notably, the low metabolic stability observed in non-human primates could possibly restrict the human application of these radioligands, especially when brain penetrating radiometabolites are formed.

**Figure 13:**
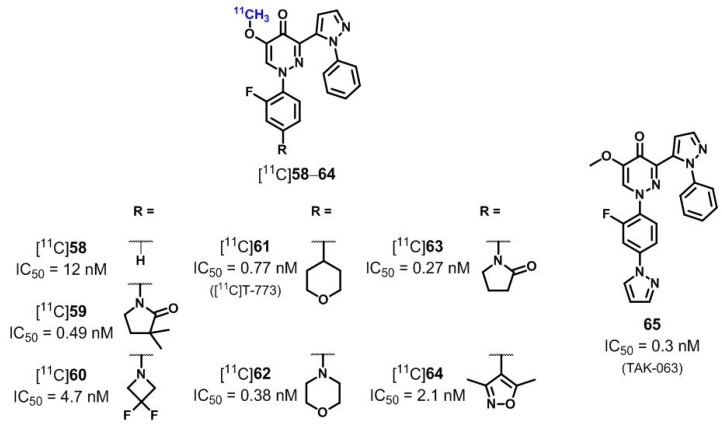
Molecular structures of ^11^C-labeled PDE10A radioligands for PET and the PDE10A inhibitor **65**.

**Scheme 19:**
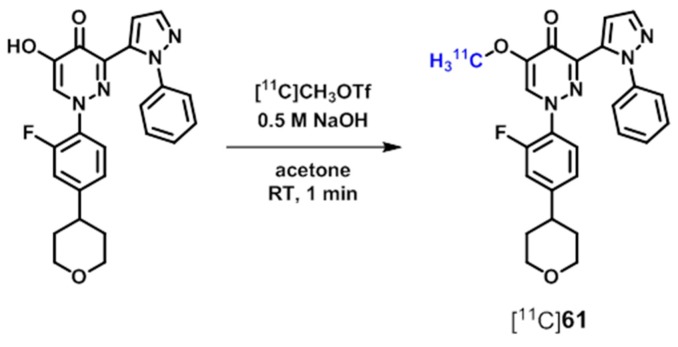
Radiosynthesis of [^11^C]**61**.

Moreover, a series of ^11^C-labeled radioligands based on a pyrazolopyridazine scaffold [189] has been reported by Stepanov *et al.* [190]. The radioligands [^11^C]**58**–**64** (Figure 13) have been prepared by *O*-alkylation of the corresponding 1,3-disubstituted 5-hydroxypyridazin-4(1*H*)-one precursors using [^11^C]methyl triflate under basic conditions [190] (Scheme 19 for [^11^C]**61**).

Characterization of these radioligands in non-human primates revealed that [^11^C]**58**–**60** and [^11^C]**62**–**64** are not suitable for PET imaging of PDE10A in the brain because of low brain uptake, moderate to fast *in vivo* degradation as well as formation of brain penetrating radiometabolites, low specific binding, and unfavorable kinetic profiles [190].

The radioligand [^11^C]**61** ([^11^C]T-773) has been stated as the most promising derivative out of this series. *In vitro* autoradiography with the tritiated analogue [^3^H]**61** ([^3^H]T-773) on brain sections of mouse displayed region-specific binding in caudate putamen, globus pallidus, nucleus accumbens, and substantia nigra while no accumulation in PDE10A knock-out animals was observed [191]. By co-incubation with various PDE10A inhibitors such as compound **22** (MP-10) and reference **61** (T-773), a considerably decreased accumulation of [^3^H]**61** in wild-type mouse and rat brain sections indicates high specific binding of this radioligand [191]. Additionally, *in vitro* autoradiography with [^3^H]**61** and [^11^C]**61** on primate and human brain sections revealed high accumulation in striatum that is comparable to the known expression pattern of PDE10A in monkey and human brain [131] and could be blocked significantly by **22** in both species [191,192].

PET imaging studies in rhesus monkeys with [^11^C]**61** revealed a high brain uptake with peak accumulation in putamen and caudate and lowest uptake in cortical regions, as well as favorable brain kinetics. [^11^C]**61** exhibited a high putamen-to-cerebellum ratio of about 5.3 at 120 min and a mean striatum-to-cerebellum ratio of 5.1 from 87 min to 123 min post injection [190,192]. Metabolism studies revealed moderate stability of [^11^C]**61** with 56% and 45% of the intact radioligand in plasma at 30 min and 90 min post injection [191], respectively, and four radiometabolites which are more polar than [^11^C]**61** [190]. PET-studies with **22** as blocking agent displayed substantial decrease in striatal uptake as well as putamen-to-cerebellum ratio and demonstrated high PDE10A-specific and reversible binding of [^11^C]**61** [192].

Moreover, [^11^C]**61** has been evaluated in PDE10A knock-out and normal mice with PET [193]. These studies showed that [^11^C]**61** is appropriate for *in vivo* quantification of PDE10A in the rodent brain and revealed significantly lower striatal protein levels in knock-out compared to control animals with %SUVs of 48.2 and 63.6 for heterozygous and homozygous knock-out mice, respectively, and 85.1 for wild-type mice between 15 and 63 min post injection [193].

Very recently, [^11^C]**61** has been applied in a PET study [194] investigating the PDE10A brain occupancy by the novel PDE10A inhibitor **65** (TAK-063, Figure 13) in non-human primates [195]. The results additionally provide evidence for the suitability of [^11^C]**61** to evaluate striatal PDE10A levels *in vivo* although unclear interactions of uptake between [^11^C]**61** and **65** in the non-target region cerebellum have been observed (decreased uptake of [^11^C]**61** after pre-treatment with **65**) [194].

In conclusion, the favorable brain kinetics, high specificity of binding and preliminary dosimetry results indicate that [^11^C]**61** might be a promising PET radioligand for *in vivo* imaging and quantification of PDE10A in the human brain.

**Figure 14:**
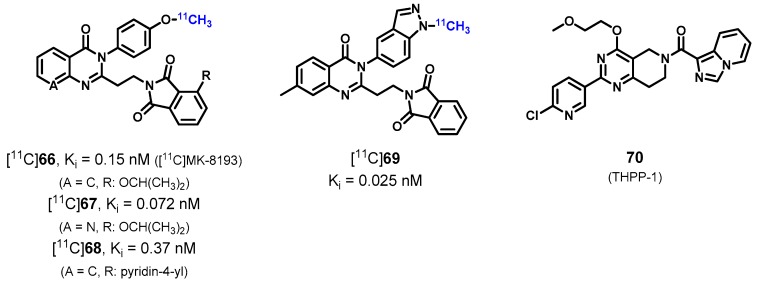
Molecular structures of ^11^C-labeled PDE10A radioligands for PET and the PDE10A inhibitor **70**.

Finally, the radioligands [^11^C]**66**–**69** (Figure 14), published by Cox and Hostetler *et al.* [196,197], have been selected from Merck high-throughput screening hits mainly on the basis of the high PDE10A-affinity of their non-radioactive analogues as well as > 27,000–100,000-fold selectivity over other PDEs. 

In baseline PET studies in rhesus monkeys, [^11^C]**66** ([^11^C]MK-8193) showed a high brain uptake with a PDE10A-specific accumulation in striatum followed by a fast wash-out phase. In comparison to [^11^C]**66**, the derivative [^11^C]**68** displayed high non-specific binding and a possibly brain penetrating radiometabolite, [^11^C]**67** showed lower specificity of binding, and [^11^C]**69** revealed slower overall elimination [196]. Development of a [^18^F]fluoroethyl analogue of [^11^C]**66** and *in vivo* evaluation of this radioligand has also been mentioned briefly without any related data published so far. Besides, further ^11^C-labeled analogues of the selective PDE10A inhibitor **70** (THPP-1, Figure 14) [198] have been studied *in vivo* that uniformly displayed poor brain uptake as also shortly reported in this article [196].

**Scheme 20:**
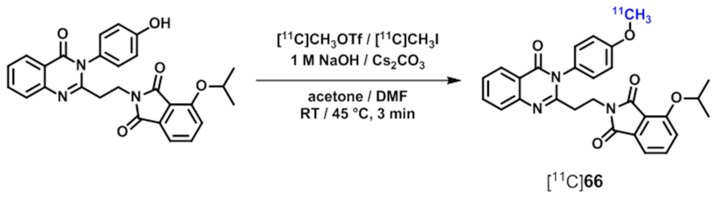
Radiosynthesis of [^11^C]**66**.

Consequently, the authors stated [^11^C]**66** to be the most promising candidate out of this series because of its favorable *in vivo* profile including brain uptake and kinetics, as well as an appropriate target-to-non-target ratio indicating high specific binding [196]. Radiosynthesis of [^11^C]**66** has been performed by *O*-alkylation of the corresponding phenol precursor using [^11^C]methyl triflate or [^11^C]methyl iodide under basic conditions [196,197,199] (Scheme 20).

*In vitro* autoradiography with the tritiated analogue [^3^H]**66** ([^3^H]MK-8193) on rat, rhesus monkey, and human brain sections displayed high and specific binding in caudate putamen and nucleus accumbens, and moderate to low binding density in hippocampus, cerebellum, brain stem, thalamus, and cerebral cortex for all three species [199]. Binding studies with [^3^H]**66** in rat, monkey, and human striatum homogenates revealed *K*_D_-values of 0.11 nM, 0.12 nM, and 0.13 nM, respectively, demonstrating high affinity of this radioligand towards PDE10A [199]. In further PET imaging studies in rhesus monkeys, a rapid and high brain uptake of [^11^C]**66** has been observed with peak accumulation in striatum at 30 min post injection (SUV ~ 2.4) followed by a fast clearance [199] which is consistent with the results described above. In contrast, PET studies in rats showed lower initial peak uptake in striatum (SUV ~ 0.7) [199]. Blocking studies after pre-treatment with **70** displayed a significantly decreased striatal uptake in both species [199] indicating high specific binding of [^11^C]**66**. Although no detailed data concerning *in vivo* stability of [^11^C]**66** have been published yet, there is no evidence for brain penetrating radiometabolites [199]. Based on these preliminary findings, [^11^C]**66** ([^11^C]MK-8193) appears to be a suitable radioligand for PET imaging of PDE10A in the brain. 

Overall, 35 novel PDE10A radioligands have been published since 2012 indicating the great interest in this enzyme as an important target in the diagnosis and treatment of several diseases. Especially for neurodegenerative and neuropsychiatric disorders, further advances in investigating the role of PDE10A were enabled by appropriate imaging agents for PET. For example, clinical PET studies clearly showed a pathological loss of striatal PDE10A in Huntington’s disease but indicated that the expression of PDE10A is not altered in schizophrenia leading to a discussion on the involvement of the PDE10A protein in schizophrenia. Furthermore, *in vivo* quantification of PDE10A in pre-clinical PET studies revealed an increase in the availability of this enzyme in both the striatum and the brown adipose tissue in obese mice pointing to an association of PDE10A with obesity. In conclusion, further evaluation of promising radioligands for imaging and quantification of PDE10A by PET will certainly result in new insights related to diverse research fields.

## 7. Summary and Concluding Remarks

The regulatory role of cyclic nucleotide phosphodiesterases in the intracellular signal transduction cascades initiated by the secondary messengers cAMP and cGMP is the key reason for the extensive development and application of numerous PDE inhibitors as therapeutics and PDE radioligands as imaging agents for PET. Various clinical indications are determined by the variant-specific expression pattern of the PDE isoenzymes within the organism and include both peripheral diseases as well as disorders of the central nervous system.

The main challenges in generating appropriate PDE radioligands are: (**1**) a high selectivity towards the particular PDE family, especially for PDE-specific detection in tissues expressing different PDE isoforms (e.g., striatal expression of PDE2A and PDE10A in the brain); and (**2**) a sufficiently high metabolic stability *in vivo* to enable a precise quantification of the respective PDE in the organ, tissue, or brain region under investigation. Furthermore, it is necessary to take into account the differences among species in the PDE isoenzyme-specific expression, distribution pattern, density, and protein structure [9,15] as well as in the metabolism [200] that may cause altered radioligand binding potency and *in vivo* stability. Thus, the radioligand evaluation in humans could lead to unexpected outcomes compared to pre-clinical studies in rodents and monkeys.

Up to now, the development of several ^11^C- and ^18^F-labeled PET radioligands for *in vivo* imaging and quantification of the PDEs 1, 2, 4, 5, 7, and especially 10 has been reported as reviewed before by Andrés *et al.* [25] in 2012 and updated herein by 49 novel radioligands (see Table 1). 

To the best of our knowledge, five of the PDE radioligands summarized in Table 1 have already entered first clinical PET studies in humans, namely [^18^F]**2** ([^18^F]PF-05270430) as a PDE2-specific imaging agent as well as the PDE10 radioligands [^18^F]**48** ([^18^F]MNI-654), [^18^F]**49** ([^18^F]MNI-659), [^11^C]**52** ([^11^C]IMA107), and [^11^C]**54** ([^11^C]Lu AE92686) for investigation of these isoenzymes in the brain. Moreover, pre-clinical *in vivo* evaluation provided evidence for additional eight PET radioligands to be promising candidates for visualization and quantification of PDE5 in lung and heart ([^11^C]**12**, [^18^F]**13**, [^11^C]**16**), and for neuroimaging of PDE10 ([^11^C]**30** ([^11^C]TZ1964B), [^11^C]**66** ([^11^C]MK-8193), [^18^F]**37**, [^18^F]**40**, [^18^F]**47** ([^18^F]AQ28A)), and further biological characterization is awaited to confirm the suitability of these compounds. Application of the other reported ^11^C- and ^18^F-labeled PDE radioligands is hampered due to low metabolic stability, poor specific binding or unfavorable kinetic profiles.

Besides the main focus on the development of PDE10 radioligands since 2010, specific radioligands for the other PDE families gain in importance as reflected by the increasing number of groups working in this field. PDE2 radioligands, for example, may be useful for PET imaging of Alzheimer’s disease as well as of highly proliferative processes associated with certain types of cancer. Regarding PET neuroimaging of PDE4, the observed low tolerability of the PDE4 protein towards structural modifications at the lead scaffold rolipram might be an explanation for the development of only one novel ^18^F-labeled derivative ([^18^F]**7**, [^18^F]MNI-617 [82,83]) since the first report by DaSilva *et al.* [23] in 1997. The situation concerning imaging of PDE5 in the brain via PET is comparable. Already 10 years ago, Jacobsen *et al.* [201] published the first radioligand for that purpose. Although there is strong evidence that PDE5 activity is related to neuroinflammatory and neurodegenerative processes as well as cognition, only one further radioligand for neuroimaging of that isoenzyme has been developed very recently by researchers from France and our group ([^18^F]**18** ([^18^F]ICF24027)) [110]. Finally, in 2015 the first two PET radioligands for imaging of PDE7, [^18^F]**20** ([^18^F]MICA-003) and [^11^C]**21** ([^11^C]MICA-005), have been reported by Thomae *et al.* [121,122] to investigate the role of this enzyme in various neurological diseases.

In conclusion, all these efforts reflect the growing interest in the development of radiolabeled isoenzyme-specific PDE ligands for diagnostic and companion imaging by PET, and enlightening findings in this area of clinical research can be expected within the next few years.

## Figures and Tables

**Table 1:** Update of novel radioligands for PET imaging of PDEs 2, 4, 5, 7, and 10 since 2012.

Phosphodiesterase	Radioligands Review Compound No. (Code No. Given)	References
**PDE2**	[^18^F]**1** ([^18^F]B-23)	[48,55]
	[^18^F]**2** ([^18^F]PF-05270430)	[48,56,57,58]
	[^18^F]**3**, [^18^F]**4**, [^18^F]**5** ([^18^F]TA3–5)	[60,61,62]
**PDE4**	[^18^F]**7** ([^18^F]MNI-617)	[82]
**PDE5**	[^11^C]**8** ([^11^C]sildenafil), [^18^F]**13**, [^11^C]**14**, [^11^C]**16**, [^11^C]**17**	[97]
	[^18^F]**18** ([^18^F]ICF24027)	[110]
**PDE7**	[^18^F]**20** ([^18^F]MICA-003), [^11^C]**21** ([^11^C]MICA-005)	[121,122]
**PDE10**	Structurally related to compound **22** (MP-10)	
	[^18^F]**26**, [^11^C]**29**	[143]
	[^11^C]**30** ([^11^C]TZ1964B), [^11^C]**31**–**33**	[157,160]
	[^18^F]**34**–**40**	[158]
	[^11^C]**42**	[161]
	Structurally not derived from compound 22 (MP-10)	
	[^18^F]**47** ([^18^F]AQ28A)	[170,172]
	[^18^F]**48** ([^18^F]MNI-654), [^18^F]**49** ([^18^F]MNI-659)	[175,176,177,178]
	[^11^C]**50** ([^11^C]IMA104), [^11^C]**51** ([^11^C]IMA106)	[142,181]
	[^11^C]**52** ([^11^C]IMA107), [^18^F]**53** ([^18^F]IMA102)	
	[^11^C]**54** ([^11^C]Lu AE92686)	[182,183]
	[^11^C]**55** ([^11^C]AMG 7980), [^18^F]**56** ([^18^F]AMG 580)	[185,186,187]
	[^11^C]**58**–**60**, [^11^C]**61** ([^11^C]T-773), [^11^C]**62**–**64**	[190,191,192,193,194]
	[^11^C]**66** ([^11^C]MK-8193), [^11^C]**67**–**69**	[196,197,199]
